# Treatment outcomes of pediatric acute myeloid leukemia in Western Kenya before and after the implementation of the SIOP PODC treatment guideline

**DOI:** 10.1002/cnr2.1849

**Published:** 2023-06-22

**Authors:** Romy E. van Weelderen, Noa E. Wijnen, Festus Njuguna, Kim Klein, Terry A. Vik, Gilbert Olbara, Gertjan J. L. Kaspers

**Affiliations:** ^1^ Pediatric Oncology, Emma Children's Hospital, Amsterdam UMC Vrije Universiteit Amsterdam Amsterdam The Netherlands; ^2^ Pediatric Oncology Princess Máxima Center for Pediatric Oncology Utrecht The Netherlands; ^3^ Child Health and Pediatrics Moi University/Moi Teaching and Referral Hospital Eldoret Kenya; ^4^ Wilhelmina Children's Hospital University Medical Center Utrecht Utrecht The Netherlands; ^5^ Pediatrics Indiana University School of Medicine Indianapolis Indiana USA

**Keywords:** Kenya, low‐ and middle‐income countries, pediatric acute myeloid leukemia, sub‐Saharan Africa, survival

## Abstract

**Purpose:**

The Pediatric Oncology in Developing Countries (PODC) committee of the International Society of Pediatric Oncology (SIOP) published a pediatric acute myeloid leukemia (AML)‐specific adapted treatment guideline for low‐ and middle‐income countries. We evaluated the outcomes of children with AML at a large Kenyan academic hospital before (period 1) and after (period 2) implementing this guideline.

**Patients and Methods:**

Records of children (≤17 years) newly diagnosed with AML between 2010 and 2021 were retrospectively studied. In period 1, induction therapy comprised two courses with doxorubicin and cytarabine, and consolidation comprised two courses with etoposide and cytarabine. In period 2, a prephase with intravenous low‐dose etoposide was administered prior to induction therapy, induction course I was intensified, and consolidation was adapted to two high‐dose cytarabine courses. Probabilities of event‐free survival (pEFS) and overall survival (pOS) were estimated using the Kaplan–Meier method.

**Results:**

One‐hundred twenty‐two children with AML were included – 83 in period 1 and 39 in period 2. Overall, 95 patients received chemotherapy. The abandonment rate was 19% (16/83) in period 1 and 3% (1/39) in period 2. The early death, treatment‐related mortality, complete remission, and relapse rates in periods 1 and 2 were 46% (29/63) versus 44% (14/32), 36% (12/33) versus 47% (8/17), 33% (21/63) versus 38% (12/32), and 57% (12/21) versus 17% (2/12), respectively. The 2‐year pEFS and pOS in periods 1 and 2 were 5% versus 15% (*p* = .53), and 8% versus 16% (*p* = .93), respectively.

**Conclusion:**

The implementation of the SIOP PODC guideline did not result in improved outcomes of Kenyan children with AML. Survival of these children remains dismal, mainly attributable to early mortality.

## 1 INTRODUCTION

Annually, there are approximately 429 000 new childhood cancer cases worldwide, but almost half are undiagnosed.^1^, ^2^, ^3^, ^4^, ^5^ One‐third of these new cases are leukemia, of which 15%–20% concern acute myeloid leukemia (AML). Since over 80% of all new cases occur in low‐ and middle‐income countries (LMICs),^1^ around 20 000 children develop AML in these countries yearly.^6^

In high‐income countries (HICs), long‐term probabilities of overall survival (pOS) of children with AML currently exceed 70%.^7^, ^8^, ^9^, ^10^ Considerably poorer rates have been reported in LMICs,^11^ in particular in sub‐Saharan Africa.^12^, ^13^, ^14^ Together with low salvage rates after relapse, high rates of treatment abandonment, early death (ED), and treatment‐related mortality (TRM) mainly explain these inferior outcomes in LMICs.^11^ Furthermore, limited resources, such as the unavailability of chemotherapeutics and essential supportive care measures (e.g., antibiotics, blood products), add to the poor outcomes.^11^ Consequently, the management of pediatric AML in LMICs is challenging.

In 2019, the Pediatric Oncology in Developing Countries (PODC) committee of the International Society of Pediatric Oncology (SIOP) published a pediatric AML‐specific adapted treatment guideline for level 2 settings in LMICs as per PODC framework definition.^6^, ^15^ Recommendations in this guideline include a prephase with low‐dose chemotherapy prior to standard treatment, either with oral etoposide combined with 6‐thioguanine and prednisolone (PrET), or with single‐agent etoposide administered orally or intravenously (i.v.), and a high‐dose cytarabine (HDAC) consolidation regimen on three consecutive days, instead of on days 1, 3, and 5. A prephase would put the leukemia in partial remission and would provide time to treat any active infections, improve the patients' clinical condition and nutritional status, and arrange financial support. It is anticipated that with this approach, the following induction therapy can be administered more safely, hopefully resulting in lower TRM and subsequent improved OS. A 3‐day consolidation course would result in shortened hematologic recovery time, lower need for platelet transfusions, and less infections, without decreasing survival.^6^

The Moi Teaching and Referral Hospital (MTRH) in Eldoret, Western Kenya, implemented the SIOP PODC treatment guideline. The aim of this study was to evaluate the outcomes of Kenyan children with AML treated before and after this implementation.

## 2 METHODS

### 2.1 Setting

Kenya is a sub‐Saharan lower‐middle‐income country in East Africa with a population of approximately 55 million.^16^ A government‐owned health‐insurance system is available, the National Hospital Insurance Fund (NHIF), which, after enrollment and activation, covers inpatient treatment costs in public health care facilities.^17^, ^18^ However, only 15% of Kenyans have NHIF and the majority pays health care out‐of‐pocket.^17^

The Shoe4Africa Children's Hospital at MTRH is one of the few public hospitals where pediatric oncology patients can be treated comprehensively. This hospital is a level 2 setting based on infrastructural and service availability characteristics as per PODC framework definition.^6^, ^15^ With over 400 daily pediatric in‐ and outpatients, Shoe4Africa is the only public children's hospital in East Africa, serving children from neighboring East African countries as well.^19^ Based on previous data, the hospital's catchment area is roughly 7 million children under 15 years of age, and around 700 new childhood cancer diagnoses are expected annually.^20^ However, according to the Shoe4Africa cancer registry, only 300 children were diagnosed with cancer in the most recent year. Oncology treatment options include chemotherapy, surgery, and since August 2021, also radiotherapy. There is a pediatric oncology ward since 2016, with currently 24 beds. A dedicated multidisciplinary pediatric oncology team is available including pediatric oncologists (2), a pediatrician (1), clinical officers (4), residents (2), nurses (19), a nutritionist (1), a social health care worker (1), and child life specialists (2).^18^

### 2.2 Study design and patients

This retrospective medical records study included patients newly diagnosed with AML at MTRH between January 2010 and December 2021 and aged 0 to 17 years. Exclusion criteria were a diagnosis of acute promyelocytic leukemia, juvenile myelomonocytic leukemia, isolated myeloid sarcoma, secondary AML, myelodysplastic syndromes, and myeloid leukemia in Down syndrome. The MTRH's Institutional Research and Ethics Committee approved this study.

### 2.3 Data collection

Patient data from our previous study on outcomes of pediatric AML at MTRH (January 2010 – December 2018) were used. In addition, the medical records of patients diagnosed with AML between December 2018 and December 2021 were obtained. A standardized case report form was filled out per patient, with information on sociodemographic and clinical characteristics, treatment response, and (dates) of events. Follow‐up phone calls to the parents/caregivers were performed in November 2022 if follow‐up information was lacking.

### 2.4 AML diagnosis, chemotherapy treatment, and supportive care

Suspected AML diagnoses were confirmed by morphological evaluation and/or flow cytometry analysis (available since 2013) of peripheral blood or bone marrow. Cytogenetic analysis was not available. After confirmed diagnosis, treatment options, including palliative care, were discussed with the patients' parents/caregivers.

Table 1 shows the chemotherapeutic treatment protocols and supportive care measures before the implementation of the SIOP PODC guideline, referred to as study period 1 (January 2010 – July 2019), and after its implementation, referred to as study period 2 (August 2019 – December 2021). The main treatment differences between the two periods were the administration of a prephase with i.v. low‐dose etoposide prior to standard treatment, an intensified induction course I, a HDAC consolidation regimen, and the expansion of supportive care measures in period 2. Intravenous etoposide as prephase regimen was chosen over PrET and oral etoposide because of its greater availability. During both periods, blood cultures were not routinely taken due to long processing times and frequent unavailability of blood culture bottles.

**TABLE 1:** Treatment protocols and supportive care measures for pediatric acute myeloid leukemia at MTRH.

Treatment	Study period 1	Study period 2
Chemotherapy		
Prephase I		
Etoposide	NA	50 mg/m^2^ i.v. 1 h, day 1–7
Prephase II^a^		
Etoposide	NA	50 mg/m^2^ i.v. 1 h, day 1–7
Induction I		
Doxorubicin	50 mg/m^2^ i.v. 4 h, day 1, 3, 5	50 mg/m^2^ i.v. 4 h, day 1, 3, 5
Cytarabine	100 mg/m^2^ i.v. 1 h, day 1–7	100 mg/m^2^ i.v. push every 12 h, day 1–7
IT therapy^b^	Doses according to the patients' age	Doses according to the patients' age
Induction II		
Doxorubicin	50 mg/m^2^ i.v. 4 h, day 1, 3, 5	50 mg/m^2^ i.v. 4 h, day 1, 3, 5
Cytarabine	100 mg/m^2^ i.v. push every 12 h, day 1–7	100 mg/m^2^ i.v. push every 12 h, day 1–7
IT therapy^b^	Doses according to the patients' age	Doses according to the patients' age
Consolidation I		
Etoposide	200 mg/m^2^ i.v. 1 h, day 1–3	NA
Cytarabine	100 mg/m^2^/day i.v. continuous, day 1–5	3 g/m^2^ i.v. 4 h every 12 h, day 1–3^c^
IT therapy^b^	Doses according to the patients' age	Doses according to the patients' age
Consolidation II		
Etoposide	200 mg/m^2^ i.v. 1 h, day 1–3	NA
Cytarabine	100 mg/m^2^/day i.v. continuous, day 1–5	3 g/m^2^ i.v. 4 hours every 12 h, day 1–3^c^
IT therapy^b^	Doses according to the patients' age	Doses according to the patients' age
Supportive care		
Antibacterial/antifungal prophylaxis	Oral cotrimoxazole thrice weekly, oral fluconazole once daily	Oral cotrimoxazole thrice weekly, oral ciprofloxacin once daily, oral fluconazole once daily
Antibacterial/antifungal empirical treatment^d^	Inconsistently available; i.v. ceftriaxone, i.v. amikacin, i.v. fluconazole, i.v. amphotericine B^e^	More consistently available; i.v. cefepime, i.v. amikacin, i.v. meropenem, i.v. fluconazole, i.v. amphotericine B^e^
Blood products	Frequently unavailable	More frequently available
Other	NA	Sodium bicarbonate mouth gurgles, nutritional support through nasogastric tube, steroid eye‐drops during consolidation therapy as conjunctivitis prophylaxis

*Note*: During study period 1, the infusion rate of doxorubicin changed from 1 to 4 h, the dose of etoposide was increased from 100 to 200 mg/m^2^, and the dose of cytarabine in the consolidation phase was lowered from 200 to 100 mg/m^2^. During both study periods, patients were started on allopurinol 10 mg/kg thrice daily 24 h prior to the start of chemotherapy. As long as tumor load was high, patients were hyperhydrated with 3000 mL/m^2^ 24 h before and after chemotherapy administration. Peripheral blood count recovery criteria were an ANC >1.0 × 10^9^/L, platelets >150 × 10^9^/L, and WBC >2.0 × 10^9^/L during study period 1 and an ANC >1.0 × 10^9^/L and platelets >75 × 10^9^/L during study period 2. Refractory and relapsed patients were referred to palliative care. Hematopoietic stem cell transplantation was not available.

Abbreviations: ANC, absolute neutrophil count; IT, intrathecal; i.v.; intravenous; MTRH, Moi Teaching and Referral Hospital; NA, not applicable; WBC, white blood cell count.

^a^ The prephase may be repeated once after one week if the patients' clinical condition remains poor. Induction I starts immediately after the prephase(s).

^b^ Methotrexate, cytarabine, and hydrocortisone.

^c^ In patients <12 months of age or <10 kg body weight, the dose was 100 mg/kg.

^d^ In the case of febrile neutropenia, which was defined as an ANC <1.0 × 10^9^/L with a body temperature >37.5°C.

^e^ Amphotericine B was administered to those patients with profound neutropenia who did not show improvement while receiving i.v. antibiotics and i.v. fluconazole.

### 2.5 Definitions and response criteria

Treatment abandonment was defined as failure to initiate or continue treatment with curative intent during the first four consecutive weeks after registration in the hospital.^21^ Complete remission (CR) was defined as <5% leukemic blasts by morphology in a bone marrow aspirate, assessed prior to consolidation therapy, with clear evidence of hematological regeneration in the bone marrow and in the peripheral blood. Refractory disease was defined as the failure to achieve CR. ED was defined as death within 42 days of treatment. TRM was defined as any death occurring after 42 days of treatment, in the absence of progressive cancer.^22^ Relapse diagnosis was based on the reappearance of leukemic blasts in the peripheral blood, or the occurrence of extramedullary disease after initial CR. Event‐free survival (EFS) was defined as the time from diagnosis until the first event, or last follow‐up. Events included induction failure (i.e., an immediate palliative care decision after the medical team's opinion on incurability, death prior to initiation of intended treatment, ED, death occurring after 42 days of treatment but prior to CR assessment, or refractory disease), treatment abandonment, death in CR, and relapse. OS was defined as the time from diagnosis until death, or last follow‐up.

The abandonment rate was calculated from the total number of included patients. The ED and CR rates were calculated from the number of patients who were initiated on chemotherapy. The relapse rate was calculated from the number of patients who achieved CR.

Patient delay was defined as the time from symptom onset to first admission at MTRH. Diagnosis and treatment delay were defined as the time from first admission at MTRH to confirmation of diagnosis and to start of treatment, respectively. These definitions are adopted from our previous study.^13^

### 2.6 Statistical analyses

Sociodemographic and clinical characteristics were described as percentages and as medians with interquartile ranges (IQR). Associations between these characteristics and study periods were evaluated using the Chi‐square or Fisher's exact test, as appropriate, for categorical variables and the Mann–Whitney *U* test for non‐normally distributed continuous variables. EFS was analyzed including induction failure and abandonment as an event at *t* = 0, and with abandonment censored at time of abandonment.^21^ The 2‐year probabilities of EFS (pEFS) and pOS were estimated with the Kaplan–Meier method and compared using the log‐rank test. If only the month and year of birth/death were known, the date of birth/death was arbitrarily set at day 1 of the specific month. In one relapsed patient who was known to have died, date of death was unknown and arbitrarily set at 1 month after the relapse date. SPSS version 28 was used for data analyses. Two‐sided *p‐*values of ≤.05 were considered statistically significant.

## 3 RESULTS

### 3.1 Sociodemographic and clinical characteristics

**FIGURE 1:**
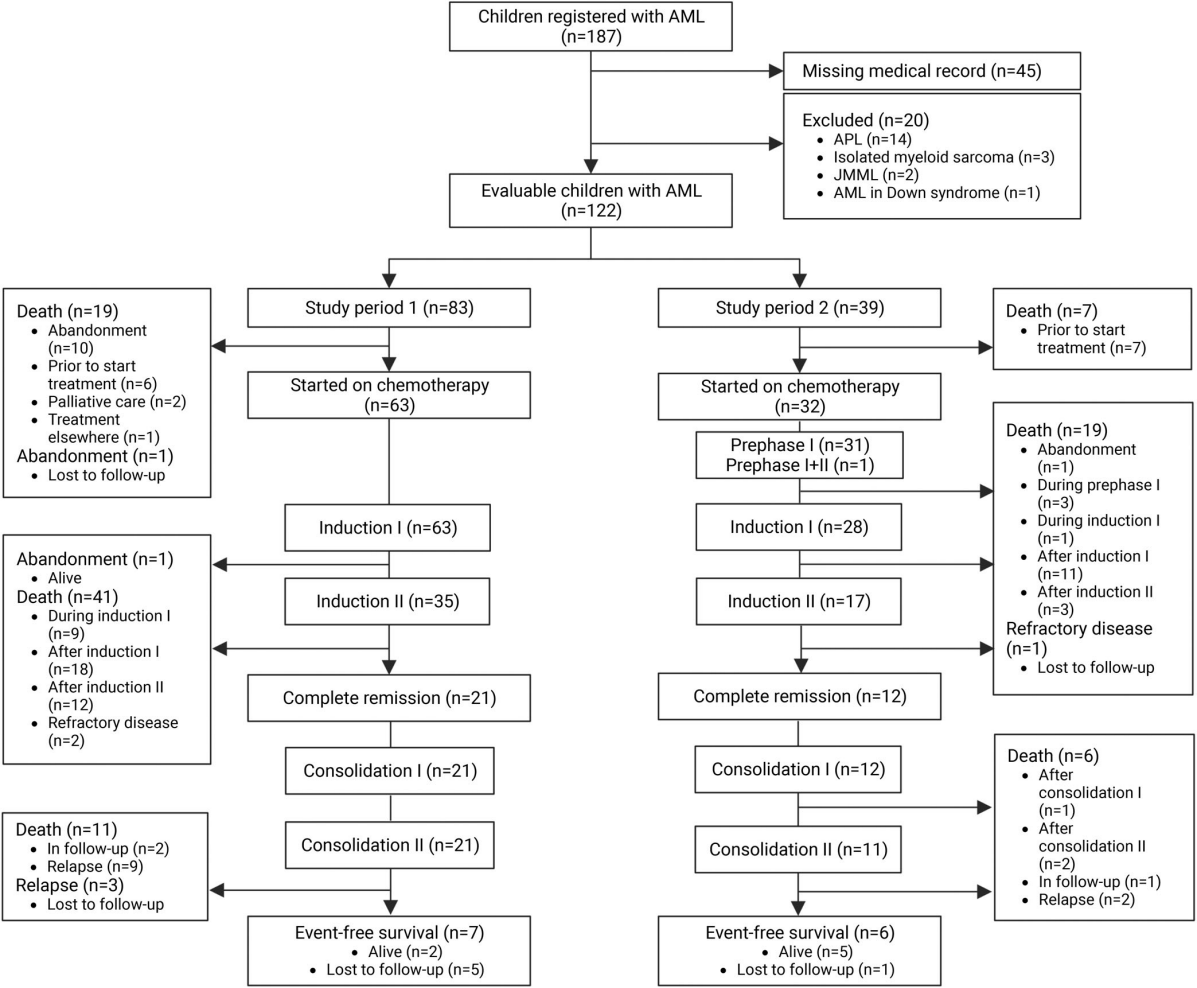
Consort flow diagram of children registered with acute myeloid leukemia at the Moi Teaching and Referral Hospital between January 2010 and December 2021. *Note*: Study periods 1 and 2 were from January 2010 to July 2019 and August 2019 to December 2021, respectively. Abbreviations: AML, acute myeloid leukemia; APL, acute promyelocytic leukemia; JMML, juvenile monomyelocytic leukemia.

One‐hundred eighty‐seven patients were registered with a diagnosis of AML between January 2010 and December 2021, of whom 20 were excluded (Figure 1). Medical records of 45 patients could not be found in the medical record office, thus were considered missing. Hence, 122 patients were included, of whom 83 in period 1 and 39 in period 2 (Figure 1).

Table 2 shows the sociodemographic and clinical characteristics of the included patients, stratified by study period. There were 69 (56.6%) males. The cohort's median age at diagnosis was 8.6 years (IQR, 5.0–11.0). Most children (*n* = 67, 54.9%) resided ≥100 km from MTRH. Nearly all patients (*n* = 113, 92.6%) were referred to MTRH from another health care facility. Of the referred patients, 47 (41.6%) were known to be diagnosed with cancer at the referring facility. At diagnosis, 56 (45.9%) patients were NHIF contributors, which increased to 77 (78.2%) patients at end of treatment. There were no statistically significant differences in the sociodemographic and clinical characteristics shown in Table 2 between the two periods (data not shown). However, the proportion of patients enrolled into NHIF at diagnosis showed a trend to be higher in period 2 (39.8% vs. 59.0%; *p* = .053).

**TABLE 2:** Sociodemographic and clinical characteristics of pediatric patients with acute myeloid leukemia diagnosed at MTRH, Eldoret (January 2010–December 2021).

Characteristics	Study period 1 *N* = 83	Study period 2 *N* = 39
Age at diagnosis, *n* (%)		
Median (IQR)	8.8 (5.9–11.0)	8.0 (3.0–11.0)
Sex, *n* (%)		
Male	47 (56.6)	22 (56.4)
Distance to MTRH, *n* (%)		
<50 km	13 (15.7)	7 (18.0)
50–100 km	24 (28.9)	10 (25.6)
≥100 km	45 (54.2)	22 (56.4)
Unknown	1 (1.2)	0 (0)
Referred from another health care facility, *n* (%)		
No	5 (6.0)	3 (7.7)
Yes	77 (92.8)	36 (92.3)
Unknown	1 (1.2)	0 (0)
NHIF status at diagnosis, *n* (%)		
No	49 (59.0)	16 (41.0)
Yes	33 (39.8)	23 (59.0)
Unknown	1 (1.2)	0 (0)
NHIF enrollment during AML treatment, *n* (%)		
No	30 (61.2)	5 (31.3)
Yes	17 (34.7)	4 (25.0)
Unknown	2 (4.1)	7 (43.8)
Symptom duration prior to first MTRH admission, *n* (%)		
<1 month	31 (37.4)	15 (38.5)
≥1 month	49 (59.0)	24 (61.5)
Unknown	3 (3.6)	0 (0)
Test to establish AML diagnosis, *n* (%)		
Peripheral blood	6 (7.2)	3 (7.7)
Bone marrow aspirate	35 (42.2)	12 (30.8)
Peripheral blood and bone marrow aspirate	27 (32.5)	24 (61.5)
Unknown	5 (6.0)	0 (0)
WBC (×10^9^/L) at diagnosis, *n* (%)		
Median (IQR)	37.4 (15.5–101.3)	37.2 (7.2–129.4)
<100	49 (59.0)	25 (64.1)
≥100	17 (20.5)	12 (30.8)
Unknown	17 (20.5)	2 (5.1)
CNS involvement present at diagnosis, *n* = 43	4 (13.3)	2 (15.4)

Abbreviations: AML, acute myeloid leukemia; CNS, central nervous system; IQR, interquartile range; MTRH, Moi Teaching and Referral Hospital; NHIF, National Hospital Insurance Fund; WBC, white blood cell count.

Most patients had experienced symptoms for ≥1 month prior to admission at MTRH (*n* = 73, 59.8%). Patient delay could not be calculated as the exact dates of symptom onset were not documented. The median diagnosis delays were 7 (IQR, 3–13) and 5 (IQR, 3–9) days, and the median treatment delays were 16 (IQR, 9.8–28.3) and 7 (IQR, 3–12.8) days in periods 1 and 2, respectively.

### 3.2 Outcomes

Figure 1 shows the consort flow diagram. Of the 122 included patients, 95 (77.9%) could be initiated on chemotherapy. Among the 63 patients who were started on chemotherapy during period 1, there were four patients who initially abandoned treatment but were readmitted and later consented for chemotherapy. Two patients abandoned during treatment; one during induction I in period 1 and one after prephase I in period 2. The abandonment rate was 19.3% (16/83) in period 1 and 2.6% (1/39) in period 2. Table 3 shows the first events of all included patients, except one patient who requested chemotherapy treatment elsewhere.

**TABLE 3:** First events among pediatric patients with acute myeloid leukemia diagnosed at MTRH, Eldoret (January 2010–December 2021).

First event	Study period 1 *N* = 82, *n* (%)	Study period 2 N = 39, *n* (%)
Treatment abandonment		
Failure to start treatment	15 (18.3)	0 (0)
Failure to continue treatment	1 (1.2)	1 (2.6)
Induction failure		
Immediate palliative care decision	2 (2.4)	0 (0)
Death prior to start treatment	6 (7.3)	7 (17.9)
Early death	28 (34.1)	14 (35.9)
Refractory disease	1 (1.2)	1 (2.6)
Death ≥42 days but prior to CR evaluation	8 (9.8)	4 (10.3)
Relapse	12 (14.6)	2 (5.1)
Death in CR		
During consolidation	0 (0)	3 (7.7)
During follow‐up	2 (2.4)	1 (2.6)
No event/Event‐free survival	7 (8.5)	6 (15.4)

*Note*: One patient is excluded from this table because of the wish to receive treatment elsewhere. Since four patients initially abandoned, which is their first event, but readmitted at MTRH and consented for chemotherapy, the numbers and percentages of patients with early death, refractory disease, and death ≥42 days but prior to CR evaluation in period 1 in this first event table do not exactly correspond with those in the text and Figure 1.

Abbreviations: CR, complete remission; MTRH, Moi Teaching and Referral Hospital.

The ED rate was 46.0% (29/63) in period 1 and 43.8% (14/32) in period 2. In period 1, most EDs occurred within the first 14 days of treatment (*n* = 20, 69.0%), whereas in period 2, most EDs occurred within 15–42 days (*n* = 11, 78.6%). The main causes of ED in period 1 were respiratory distress not further specified (*n* = 10), sepsis (*n* = 10), and excessive hemorrhage (*n* = 4), whereas in period 2, the main cause of ED was sepsis (*n* = 9) and none of the patients died of excessive hemorrhage. Ten patients died after 42 days of treatment but prior to CR evaluation in period 1 and four in period 2, with the most common cause of death being sepsis.

The CR rate was 33.3% (21/63) in period 1 and 37.5% (12/32) in period 2. All patients who achieved CR in period 1 completed treatment, compared to nine of the 12 patients who achieved CR in period 2. Causes of death of the three patients who died during consolidation in period 2 were sepsis (*n* = 2) and malaria (*n* = 1). Two patients from period 1 and one patient from period 2 expired during the follow‐up period after treatment completion. Their relapse status and causes of death could not be traced out. The TRM rate was 36.4% (12/33) in period 1 and 47.1% (8/17) in period 2.

The relapse rate in period 1 was 57.1% (12/21) and 16.7% (2/12) in period 2. At last follow‐up, seven of the 63 patients treated in period 1 and six of the 32 patients treated in period 2 were event‐free, with median follow‐up duration from completion of treatment of 5.8 months (range, 0.3–84.5) and 21 months (range, 4.0–34.4), respectively.

**FIGURE 2:**
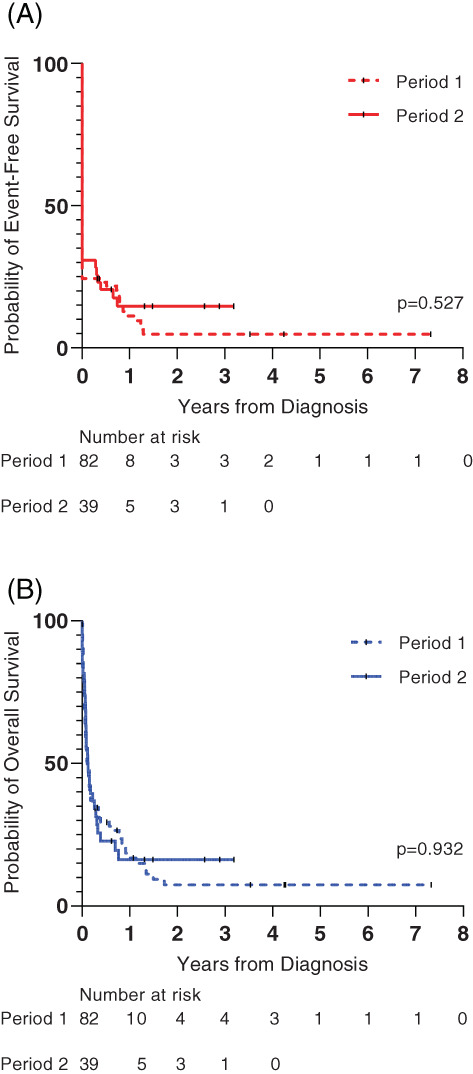
Kaplan–Meier estimates of event‐free survival (A) and overall survival (B) of children with acute myeloid leukemia treated at MTRH. *Note*: Study periods 1 and 2 were from January 2010 to July 2019 and August 2019 to December 2021, respectively. Abbreviation: MTRH, Moi Teaching and Referral Hospital.

The overall cohort's 2‐year pEFS and pOS were 7.8% (*SE*, 2.7) and 10.1% (*SE*, 3.2), respectively. The 2‐year pEFS and pOS did not differ statistically significantly between study period 1 and 2, whilst these outcomes seemed to be better in study period 2 (EFS: 14.7% [*SE*, 5.8] vs. 4.6% [*SE*, 2.6], *p* = .53; OS: 15.9% [*SE*, 6.1] vs. 7.5% [*SE*, 3.4], *p* = .93; Figure 2).

If the patients who abandoned treatment were censored at the time of abandonment, the 2‐year pEFS increased to 8.0% (*SE*, 4.3) in period 1 and 15.9% (*SE*, 6.1) in period 2 (*p* = .45). If the five patients who had an event but were lost to follow‐up were assumed to be deceased, the 2‐year pOS dropped to 5.9% (*SE*, 2.8) in period 1 and to 14.7% (*SE*, 5.8) (*p* = .86) in period 2.

## 4 DISCUSSION

This study evaluated outcomes of Kenyan children with AML before and after implementing the SIOP PODC pediatric AML‐specific adapted treatment guideline. There was no significant difference in the 2‐year pEFS (5% vs. 15%; *p* = .53) and pOS (8% vs. 16%; *p* = .93) after the implementation of the guideline.

The prephase was recommended to lower TRM and improve OS. However, the TRM rate was 47% in period 2 and 36% in period 1, and the 2‐year pOS was not statistically significantly different (16% vs. 8% in period 1). Our results cannot be compared with those achieved by others as, to the best of our knowledge, no previous studies evaluated the efficacy of the guideline.

ED rates were remarkably high in both periods. Induction course I included intensified cytarabine during period 2, thereby possibly negating the potential benefit of the prephase. It may be considered to administer standard two or three repetitive prephase cycles, since all patients in period 2 except one received only one prephase cycle, or to administer a low‐dose induction course I. The aim hereof is to lower toxicity. These alternatives are also described in the SIOP PODC guideline.^6^

Our findings highlight the challenging balance between anti‐cancer treatment and supportive care measures, as a structured approach of supportive care is key to reduce morbidity and mortality in these resource‐limited settings. In LMICs, bacterial and fungal infections during neutropenic episodes are the main cause of mortality.^23^ Despite the expansion of antibacterial prophylaxis and the increased availability of antibiotics in our setting, many patients died of sepsis. It has to be acknowledged that sepsis was most often a clinical diagnosis, as blood cultures were not routinely taken. Several factors might have contributed to this high infection‐related mortality rate. First, the lack of prompt blood culture results hampered the adequate administration of antibacterial therapy, as the causative pathogens and possible multidrug resistance patterns remained unidentified. Second, in febrile neutropenic patients, immediate administration of antibiotics remained challenging due to overcrowding and lack of personnel. For instance, one nurse takes care of more than 15 patients (dr. F. Njuguna, June 2022). Finally, sharing of beds and the lack of isolation room capacity contributed to this infection risk. A study on pediatric acute leukemia in a LMIC setting also reported a high mortality rate (41%), mostly caused by infections (65%). Improved supportive care measures in that setting significantly reduced overall mortality rates to 29%.^24^ This supports the thought that addressing infection prevention and control may be the “low‐hanging fruit” with the greatest effect on outcome. Supportive care recommendations were, however, beyond the scope of the SIOP PODC guideline.^6^

Other essential aspects of supportive care are adequate availability of blood products and nutritional support. At MTRH, active recruitment of blood donors has led to a more stabilized blood product availability and will remain an important focus. Regarding nutritional support, nasogastric tubes were often not placed in patients with mucositis due to parental resistance who associate it with their child being palliative (dr. F. Njuguna, June 2022). This underscores the importance of adequate education of parents and nurses.

It is potentially promising that, though not statistically significant, the 2‐year pEFS and pOS were somewhat higher in period 2 than in period 1. This could be attributed to the intensified and condensed consolidation regimen in period 2, thereby preventing relapses (relapse rate: 17% in period 2 vs. 57% in period 1).

To achieve cure, early AML diagnosis and initiation of treatment are highly important. Yearly, the number of new AML diagnoses at MTRH increases, which, we believe, is the result of multiple efforts. First, the initiation of training of healthcare workers, for example through our Project ECHO cancer awareness program established in 2020, led to better recognition of AML at the referring facilities.^25^, ^26^ Second, children with suspected AML at the general pediatric ward have been reviewed faster, since the number of pediatric oncologists in training at MTRH increased. Third, as flow cytometry became more available with rapid results, more precise diagnosis and earlier initiation of treatment were possible. Fourth, patients without NHIF at diagnosis are currently actively guided in the enrollment process by the medical staff of MTRH, leading to an increased NHIF enrollment rate. This increased rate, together with the hospitalization of patients during the entire course of treatment, may have contributed to the decrease in abandonment rate from 19% in period 1 to 3% in period 2. Together, all these efforts have led to a decrease in diagnosis and treatment delays.

This study contributes to the scarce literature on outcome of pediatric AML in LMICs, particularly in sub‐Saharan Africa. Our survival rates remain dismal and are in striking contrast to those reported in HICs, where the current 5‐year pEFS and pOS exceed 50% and 70%, respectively.^8^, ^9^, ^27^ Our rates are also inferior compared to those reported in other LMICs, like India, Pakistan, Morocco, and El‐Salvador, where the 4‐ to 5‐year pEFS and pOS range from 24%–30% and 27%–42%,^28^, ^29^, ^30^, ^31^ respectively, treated with a similar therapy backbone as ours. From sub‐Saharan Africa, aside from Kenya, only one Tanzanian study is available, wherein all children with AML died within one year after diagnosis.^12^ Additionally, one Ugandan abstract on childhood AML reported a CR rate of 56% (9/16), with quite a similar induction therapy and supportive care regimen as in period 2 of our study.^14^

This study also contributes to the evidence on the implementation of the SIOP PODC pediatric AML‐specific adapted treatment guideline^6^ and may serve as a valuable example for other LMICs. To validate the proposed expert‐opinion based recommendations, this guideline needs to be implemented in LMICs and subsequently evaluated. Despite the well‐known obstacles associated with adjusting treatment regimens to LMIC settings,^6^ the implementation of this guideline at MTRH was easy and uncomplicated.

Limitations of this study are its retrospective design and the relatively small sample size, especially in period 2. This was unfortunately due to missing files. The efficacy of the SIOP PODC guideline could have been studied better if these files were available. The absence of these files also highlights the frailty of a resource‐limited setting, wherein often there is no adequate data registration system. Besides, patients older than 15 years of age may not have been registered in the pediatric cancer registry, since they were usually treated at the adult oncology ward. Currently, an online data registration system is being implemented at MTRH, hopefully improving the data registration process.

In conclusion, in this setting, with the supportive care provided, the SIOP PODC guideline did not improve outcome of Kenyan children with AML. Nonetheless, this study shows that it is feasible to implement this guideline in a sub‐Saharan African LMIC. Survival remains dismal, primarily because of a high ED rate caused by sepsis. Future focus at MTRH should be on adequate supportive care measures, especially infection prevention and control, since this might be the “low‐hanging fruit” to improve OS. Antibiotic stewardship should be improved, by obtaining blood cultures more routinely. These cultures should be studied in order to gain more insight into the causative pathogens and resistance patterns, so that targeted antibiotics can be initiated promptly. Education of the parents and nurses on the importance of nasogastric feeding will also be an important aspect. Additionally, future focus should be on the optimization of prephase and induction therapy. This may include prolongation of the prephase and/or administering a low‐dose induction course I. Currently, there are plans to build the first pediatric oncology hospital in sub‐Saharan Africa at MTRH with a 100‐bed capacity. Hopefully, all these efforts will ultimately result in an improved survival of children with AML in sub‐Saharan Africa.

## AUTHOR CONTRIBUTIONS

**Romy van Weelderen:** Conceptualization (equal); data curation (equal); formal analysis (lead); investigation (lead); methodology (equal); project administration (lead); validation (lead); visualization (lead); writing – original draft (lead); writing – review and editing (lead). **Noa Esther Wijnen:** Conceptualization (equal); data curation (equal); formal analysis (lead); investigation (lead); methodology (equal); project administration (lead); validation (lead); visualization (lead); writing – original draft (lead); writing – review and editing (lead). **Festus Njuguna:** Conceptualization (equal); methodology (equal); resources (lead); supervision (lead); validation (equal); writing – review and editing (equal). **Kim Klein:** Conceptualization (equal); supervision (equal); writing – review and editing (equal). **Terry A Vik:** Conceptualization (equal); supervision (equal); writing – review and editing (equal). **Gilbert Olbara:** Conceptualization (equal); resources (equal); supervision (equal); writing – review and editing (equal). **Gertjan Kaspers:** Conceptualization (equal); supervision (lead); writing – review and editing (equal).

## CONFLICT OF INTEREST STATEMENT

The authors have no conflict of interest to disclose.

## ETHICS STATEMENT

We declare that the work submitted to *Cancer Reports* has been done in accordance to the COPE principles, and that it has been performed in an ethical and responsible way, with no research misconduct, which includes, but is not limited to data fabrication and falsification, plagiarism, image manipulation, unethical research, biased reporting, authorship abuse, redundant or duplicate publication, and undeclared conflicts of interest.

## Data Availability

The data that support the findings of this study are available from the corresponding author upon reasonable request.
